# Dysfunctional Breathing in Children: A Literature Review

**DOI:** 10.3390/children11050556

**Published:** 2024-05-06

**Authors:** Georgia Karkouli, Konstantinos Douros, Dafni Moriki, Paraskevi Moutsatsou, Ioanna Giannopoulou, Eirini Maratou, Despoina Koumpagioti

**Affiliations:** 1 Allergology and Pulmonology Unit, 3rd Pediatric Department, National and Kapodistrian University of Athens, 12462 Athens, Greece; karkgogo@otenet.gr (G.K.); dmoriki@med.uoa.gr (D.M.); 2 Department of Clinical Biochemistry, National and Kapodistrian University of Athens, 12462 Athens, Greece; pmoutsatsou@med.uoa.gr (P.M.); maratou@hotmail.com (E.M.); 3 Department of Psychiatry, National and Kapodistrian University of Athens, 12462 Athens, Greece; igianno@med.uoa.gr; 4 Department of Nursing, National and Kapodistrian University of Athens, 11527 Athens, Greece; dkoumpagioti@nurs.uoa.gr

**Keywords:** dysfunctional breathing, breathing pattern disorders, induced laryngeal obstruction, exercise-induced laryngeal obstruction, vocal cord dysfunction, children

## Abstract

Dysfunctional breathing (DB) describes a respiratory condition that is mainly characterized by abnormal breathing patterns, affecting both children and adults, often leading to intermittent or chronic complaints and influencing physiological, psychological, and social aspects. Some symptoms include breathlessness; dizziness; palpitations; and anxiety, while its classification lies in breathing pattern disorders and upper airway involvement. Its prevalence among the pediatric population varies with a female overrepresentation, while the existence of comorbidities in DB, such as asthma, gastro-esophageal reflux, nasal diseases, and anxiety/depression, frequently leads to misdiagnosis or underdiagnosis and complicates therapeutic approaches. The basic diagnostic tools involve a detailed history, physical examination, and procedures such as structured light plethysmography, cardiopulmonary exercise testing, and laryngoscopy when a laryngeal obstruction is present. The management of DB presumes a multidimensional approach encompassing breathing retraining, disease-specific advice through speech and language therapy in the presence of laryngeal obstruction, psychotherapy for fostering self-efficacy, and surgical therapy in a structural abnormality. The current review was developed to provide a summary of classifications of DB and epidemiological data concerning the pediatric population, comorbidities, diagnostic tools, and therapeutic approaches to enhance the comprehension and management of DB in children.

## 1. Introduction

Dysfunctional breathing (DB) describes a respiratory condition that is mainly characterized by abnormal breathing patterns and affects both children and adults, often leading to intermittent or chronic complaints [1,2,3]. It appears that biochemical (e.g., end-tidal CO_2_) and biomechanical aspects (e.g., thoracic movements); respiratory symptoms (e.g., dyspnea); and physiological (e.g., cardiovascular effects), psychological (e.g., depression and anxiety), and social aspects (e.g., health-related quality of life) are primarily linked with the pathology and the severity of DB [4]. Symptoms may include breathlessness; chest pain/tightness; dizziness; headache; palpitations; tingling; tiredness; hyperventilation; reduced exercise tolerance; and anxiety [2]. Many breathing patterns could appear as a physiological response to disease, but when no organic abnormalities exist, these patterns can be deemed pathological [5]. Though DB can manifest independently, it is often observed in individuals with concurrent respiratory issues, for instance asthma. The symptoms of DB closely resemble those of asthma, leading to misdiagnoses and overprescriptions of asthma medications. Nevertheless, DB can be distinguished from other breathing conditions through proper history taking, assessment, and diagnostic tests [6].

Despite the fact that DB is described more extensively in adults, its impact on children is equally significant [1]. However, there is no consensus regarding the definition of DB, while most patterns documented in the literature are not applicable to the pediatric population. Additionally, there is no gold standard for its diagnosis. Dysfunctional breathing poses a global challenge for healthcare professionals due to the difficulties in its identification, resulting in frequent insufficient or incorrect diagnoses in clinical practice. This negatively impacts patients’ health-related quality of life [3,7]. We aimed to review the existing literature in order to provide a summary of classifications of DB and epidemiological data concerning the pediatric population, comorbidities, diagnostic tools and therapeutic approaches to enhance the comprehension and management of DB in children.

## 2. Materials and Methods

**Figure 1:**
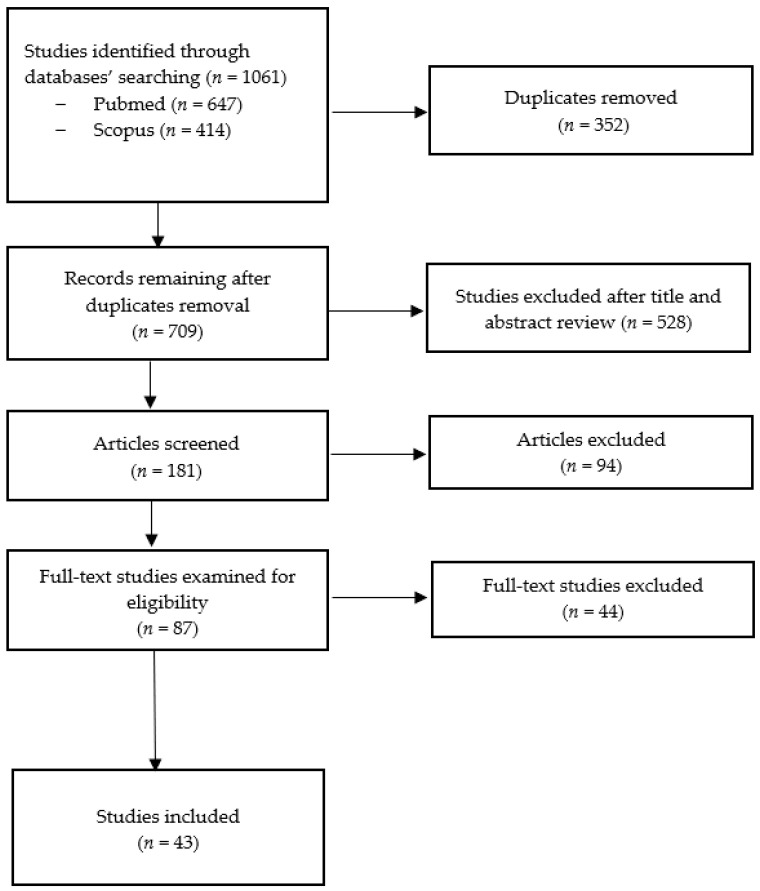
Flow diagram for study selection process.

An extensive literature review was performed using the PubMed and Scopus databases, searching for articles published from inception to January 2024, using the keywords “Dysfunctional breathing”, “Breathing pattern disorders”, “Induced laryngeal obstruction”, “Exercise-induced laryngeal obstruction”, “Vocal cord dysfunction”, and “Children”. A total of 1061 records were identified and, after removing duplicates, the remaining papers (*n* = 709) were screened for eligibility. Through a title and abstract review, 181 studies were retrieved and further examined. After a full text articles assessment, 43 studies were included in this review (see Figure 1). The study selection process was undertaken by two researchers, and articles for which there was unanimous agreement among the researchers were included in the review.

## 3. Definition and Classifications of Dysfunctional Breathing

The determination of the definition of a clinical entity and the identification of its clinical findings contribute significantly to its timely diagnosis and therapeutic management. However, the current lack of clarity on the usage of the term DB and the utilization of various terms to describe the same condition have impeded the comprehension of DB, making it challenging to objectively identify. 

According to Barker and Everard, DB is defined as “an alteration in the normal biomechanical patterns of breathing, leading to intermittent or chronic respiratory and/non-respiratory symptoms” [3]. Furthermore, they categorized DB into two types: thoracic DB (T-DB), characterized by changes in respiratory muscle activity patterns, which could or could not be linked with hyperventilation, and extra-thoracic DB (ET-DB), which occurs when there is upper airway involvement (such as paradoxical vocal cord dysfunction (pVCD)), alongside a pattern of disordered breathing. The sub-categories of T-DB and ET-DB include functional DB, which happens when normal relaxed diaphragmatic breathing is substituted with a condition where the respiratory pump relies mainly on the upper chest wall and accessory muscles (e.g., pattern disordered breathing, VCD), and structural DB, which is associated with anatomical and neurological abnormalities, such as unilateral cord palsy and subglottic stenosis [3]. A few years later, Barker et al. introduced a similar approach to DB, dividing it into two components: breathing pattern disorders (BPD) and inducible laryngeal obstruction (ILO), also known as exercise-induced laryngeal obstruction (EILO), when the obstruction is triggered by exercise. Breathing pattern disorders include the Hyperventilation Syndrome (HVS) and the subcategories of BPD, while in ILO, VCD and supraglottic collapse are included. The dysfunction of each component may manifest separately, or they may be closely interconnected and exist concurrently [1].

Boulding et al. proposed a classification of five categories of abnormal breathing patterns in adults, using recordings of quiet tidal breathing during rest, subsequently followed by maximum expiration and then inspiration: hyperventilation syndrome, which is mainly correlated with the demonstration of hypocapnia and respiratory alkalosis; periodic deep sighing, often characterized by sighing and irregular breathing patterns; thoracic dominant breathing, which happens when there is a primary use of the upper thorax with an absence of lateral costal expansion; forced abdominal expiration, which is observed with inappropriate and excessive abdominal muscle contraction in support of expiration; and thoraco-abdominal asynchrony caused by a postponement between ribcage and abdominal contraction [5]. Table 1 summarizes the classifications of DB as proposed by different authors.

## 4. Epidemiology of Dysfunctional Breathing in Pediatric Population

Determining the prevalence of DB poses a challenge due to overlapping symptom presentations and distinct diagnostic approaches. This challenge is further compounded by the fact that each sub-category is typically examined independently, and there are limited data available for children. A plausible explanation is that the identification of DB is demanding due to the resemblance of its symptoms with other respiratory conditions, as the potential coexistence of DB with these disorders further complicates the diagnostic process [1]. Table 2 depicts the main characteristics of selected studies focused on dysfunctional breathing in the pediatric population.

A retrospective, cross-sectional study was performed to gain a deeper understanding of the characteristics of children with DB, 66% of whom were female and 26% were overweight and in a poor physical condition. The most documented symptoms were breathlessness (76.6%), chest pain/tightness (51.2%), and dizziness (49.8%), while 52% of the subjects had a Nijmegen questionnaire (a questionnaire for detecting DB) score of ≥23, mainly female participants (59.8%) [2]. 

Regarding HVS, a study by D’Alba et al. investigated the prevalence of symptoms suggestive of HVS among adolescents with or without asthma. The study found that 6.2% of patients had a Nijmegen questionnaire score of ≥23, indicative of HVS. Moreover, girls exhibited a higher prevalence than boys (*p* < 0.001), and subjects with asthma were ten times more likely to exhibit symptoms indicative of HVS (25%) than non-asthmatic adolescents (2.5%). The Nijmegen questionnaire score was significantly higher in individuals with lifetime asthma (*p* < 0.001), current episodic asthma (*p* < 0.05), and current active asthma (*p* < 0.001) compared to those without asthma [8]. Gridina et al. investigated the prevalence of HVS in children and adolescents in a region of France and found a percentage of 21% of the evaluated population with HVS, with an overrepresentation of girls (24.7%) and 6.7% of participants reported as having asthma. Among asthmatic children, 55% had HVS, compared to 18.6% of non-asthmatic children (*p* = 0.0003). Additionally, asthmatic children with HVS presented a higher frequency of symptoms compared to non-asthmatic children [9].

An observational study involving respiratory outpatients aged 0–16 years reported the following findings: 16% of patients exhibited extrathoracic DB (ILO), 10% had thoracic DB (7% BPD, 1% hyperventilation, and 2% sighing tics), and 11% had asthma along with DB [10]. In a cross-sectional study in children with asthma, 5–18-year-olds had a 5.3% prevalence of DB, with a high prevalence among females (*p* < 0.002). Poor asthma control was more prevalent in subjects with DB than in those without (OR 19.3, 95% CI 3.14–430.70; *p* < 0.0001), while the median Asthma Control Questionnaire score in participants with DB was higher (median (range) 2.00 (1.50–3.17)) than in those without (0.50 (0.17–1.17); *p* < 0.001) [11]. Vahlkvist et al. conducted a cohort survey to assess the prevalence of DB among asthmatic children in a pediatric outpatient clinic and evaluate its effect on asthma control. The study found that the prevalence of DB was 18%, with a significant overrepresentation of females (*p* < 0.01). Additionally, the Forced Expiratory Volume in one second percentage (FEV_1_%) was higher in the DB group (mean (SD): 89.4 (9.0) vs. 85.7 (11.8), *p* < 0.02). The Asthma Control Questionnaire (ACQ) score (median (range): 2.0 (0–4) vs. 0.6 (0–3.4), *p* < 0.01) and the use of beta2 agonist (median (range): 2 (0–56) vs. 0 (0–20) puffs/week, *p* < 0.01) were also higher in the DB group [12]. 

Newson and Elias audited the health reports of 18 participants with BPDs, with a median age of 14 years and a female overrepresentation (14/18). The most prevalent comorbidity was asthma (16 patients) and the most common BPD was ILO (16 patients, with EILO in 15), followed by HVS in 7 patients, psychogenic cough in 8 patients, and dysphonia in 1 patient. The frequent inducers of BPD symptoms were exercise (16 patients), anxiety (14 patients), and emotion and stress (11 patients). Significant school absenteeism (ranging from 2 weeks to 24 months), delays in BPD diagnosis (range: 1–24 months, median: 6 months), and emergency calls for 14 patients were observed in the majority of the subjects [13].

In recent years, amidst the COVID-19 pandemic, long COVID patients diagnosed with newly developed dysfunctional breathing after SARS-CoV-2 infection have experienced a significant symptom load, low quality of life, and important functional consequences. Particularly, in a recent adult study aimed at describing symptoms, functional outcomes, and quality of life using both subjective and objective measures in a large case series study including long COVID patients with DB, hyperventilation, periodic deep sighs/erratic breathing, and a combination of DB types were identified in 20.8%, 47.1%, and 33.3% of participants, respectively. Following dyspnea, the most prevalent symptoms based on the Nijmegen scale with a cutoff of ≥3 involved faster/deeper breathing (75.6%), palpitations (63.8%), sighs (48.7%), being unable to breathe deeply (46.3%), and yawning (46.2%). The median Nijmegen and Hospital Anxiety and Depression Scale scores were 28 and 16.5, respectively [14]. In a study of adolescents with confirmed SARS-CoV-2 infection, it was observed that dysfunctional respiratory symptoms were reported at a mean of 4.15 (±4.24, min 1–max. 20) weeks after infection, and all subjects mentioned respiratory symptomatology during exercising, with an additional 32% experiencing symptoms while resting. In total, 16% of the participants primarily had symptoms of ILO, 44% presented typical thoracic dominant breathing with insufficient ventilation, and 40% indicated an overlap of these two causes, while 8% noted emotional stress as a stimulus [15].

Johansson et al. investigated the prevalence of exercise-induced bronchoconstriction (EIB) and EILO in a general adolescent population. They estimated that the prevalence of EILO was 5.7% (95% CI 0.01% to 11.4%) among the total of 3838 adolescents enrolled in the study, with no gender difference observed (*p* > 0.99) [16]. Shay et al. conducted a retrospective chart review of children and young adults with EILO. They found that the majority of patients were females (81.3%), with 93% of them participating in organized sports. Additionally, 59.8% of the subjects had a prior diagnosis of asthma, and most of those individuals had an unsuccessful asthma treatment. Paradoxical vocal fold motion was recorded in 78.4% of participants, and 23.4% exhibited supraglottic collapse. The most commonly reported symptoms included dyspnea (93.8%), wheezing/stridor (78.6%), and throat tightness (48.2%) [17].

**Table 2:** Characteristics of selected studies focused on dysfunctional breathing in children.

Author	Country	Study Design	Sample Characteristics	Classification of DB	Diagnosis of DB	Results
Trompenaars et al. [2]	The Netherlands	Cross-sectional	201 participants, mean age (years) ± SD: 13.9 ± 2.3	Not mentioned—symptoms suggestive of DB	Symptoms, NQ, evaluation at rest and during exercise (Bruce treadmill test or bicycle test), breathing pattern	Most frequent reported symptoms: breathlessness, chest pain/tightness, dizziness. NQ ≥ 23 for 52% of the participants, most females.
D et ‘Alba et al. [8]	Italy	Cross-sectional	760 participants, 11–14 years	HVS	NQ	Symptoms of HVS: 10 times more prevalent in asthmatic adolescents (25%). NQ ≥ 23 for 6.2% of the participants, mostly females. Higher NQ score in lifetime asthma (*p* < 0.001), current episodic asthma (*p* < 0.05), and current active asthma (*p* < 0.001).
Gridina et al. [9]	France	Cross-sectional	300 participants, 1–17 years	HVS	SHAPE questionnaire	21% of participants had HVS, with girls’ overrepresentation and 6.7% of asthmatics. Among asthmatics, 55% of them had HVS versus non-asthmatics (*p* = 0.0003).
Pedersen et al. [10]	Switzerland	Observational	214 participants, 0–16 years	Extrathoracic DB, thoracic DB	Symptoms, body plethysmography, exercise-challenge test, spirometry, bronchodilator test, bronchial challenge test	16% with extrathoracic DB (ILO), 10% with thoracic DB and 11% asthma along with DB.
De Groot et al. [11]	Denmark	Cohort	206 participants with asthma, 5–18 years	Not mentioned—symptoms suggestive of DB	NQ	11% of asthmatic children had DB (most females). Poor asthma control was more prevalent in children with DB (*p* < 0.0001).
Vahlkvist et al. [12]	Italy	Case–control	363 participants, median age: 18.8 years	Not mentioned—symptoms suggestive of DB	NQ	18% of participants had DB, with females’ overrepresentation. FEV_1_% (*p* < 0.02), ACQ score (*p* < 0.01) and the use of beta2 agonist (*p* < 0.01) were higher in the DB group.
Newson and Elias [13]	Singapore	Retrospective	18 participants, median age: 14 years	ILO—EILO, HVS, psychogenic cough, and dysphonia	History, symptoms, triggers, psychosocial history, and anxiety patterns	ILO: (16 patients, 15 with EILO), HVS: 7 patients, psychogenic cough: 8 patients, and dysphonia: 1 patient. Common comorbidity: asthma (16 patients). Inducers of BPD symptoms: exercise (16 patients), anxiety (14 patients), and emotion and stress (11 patients).
Johansson et al. [16]	Sweden	Cross-sectional	146 participants, mean age: 14.2 years	EILO	Continuous laryngoscopy during exercise	5.7% of the participants had EILO, with no gender difference (*p* > 0.99).
Shay et al. [17]	USA	Retrospective chart review	112 participants, mean age of symptoms onset (years) ± SD: 13.8 ± 3.3, mean age at diagnosis: 15.4	EILO	Flexible laryngoscopy, history	78.4% of participants had paradoxical vocal fold motion and 23.4% supraglottic collapse. Most commonly reported symptoms: dyspnea (93.8%), wheezing/stridor (78.6%), and throat tightness (48.2%). 59.8% had prior diagnosis of asthma, most with failed asthma treatment.

DB: dysfunctional breathing, NQ: Nijmegen Questionnaire, HVS: hyperventilation syndrome, SHAPE: Hyperventilation Syndrome Ambroise-Pare’ Enfant questionnaire, FEV_1_: Forced Expiratory Volume in 1 s, ACQ: Asthma control questionnaire, ILO: inducible laryngeal obstruction, EILO: exercise-inducible laryngeal obstruction, BPD: breathing pattern disorders.

## 5. Dysfunctional Breathing and Comorbidities

Since DB symptoms are unspecified, a thorough method for differential diagnosis is crucial in order to verify the implementation of appropriate management [18]. It is certain that comorbidities interact with DB, and they mutually influence each other. Their coexistence complicates both diagnosis and therapeutic approaches. The most frequent comorbidities in DB are asthma, gastro-esophageal reflux, nasal diseases, and anxiety/depression [1,18]. 

Dysfunctional breathing is linked with asthma morbidity through various potential mechanisms, encompassing breathing pattern disorders induced by anxiety, a heightened perception of subsequent symptoms, airway cooling and drying resulting from hyperventilation-induced hyperresponsiveness, and a direct influence of emotional stimuli on airway constriction via cholinergic pathways [19]. In adults, DB impacts 24–29% of individuals with asthma and 30–64% of those dealing with difficult asthma, while there is frequent relationship with ILO/VCD [20,21]. The prevalence of asthma with DB in pediatric population ranges from 5% to 18% [10,11,12], while DB has been associated with poorer asthma control and an increased utilization of beta2 agonists, and DB may potentially constitute a contributing factor in difficult-to-treat asthma [12]. 

Gastro-esophageal Reflux Disease (GERD) may be correlated with VCD, as 18% of adult patients with VCD are reported to have an underlying GERD. Nonetheless, the real connection between GERD and VCD remains uncertain, as the laryngoscopic findings utilized for diagnosing laryngopharyngeal reflux are nonspecific [22,23]. Powell et al. evaluated the demographic and videolaryngoscopic features of juveniles with paradoxical VCD and found that 95% of the subjects demonstrated arytenoid and interarytenoid edema with pachyderma frequently encountered in GERD [24]. Furthermore, it seems that, among infants, VCD has been strongly related to GERD, since, in a study of nine infants with a laryngeal dyskinesia history, the existence of inspiratory vocal cord adduction was observed and eight out of nine infants had a documented history of GERD, showing symptomatic improvement after treatment [25]. 

Nasal disease frequently appears as a complicating comorbidity in ILO and BPD, however, treating nasal disease can enhance not only nasal breathing, but likewise alleviate the symptoms associated with ILO and BPD [18,26,27]. In particular, chronic rhinosinusitis exacerbates comorbidity in vocal cord dysfunction (VCD). It leads to laryngeal sensitization, wherein the larynx exhibits exaggerated responses to triggers that would typically be harmless in individuals without symptoms, thus resulting in laryngeal hyperresponsiveness [28]. Moreover, nasal obstruction encourages mouth breathing, leading to laryngeal dryness and heightened irritability, provoking bypassing the usual nasal breathing which ordinarily causes cordal opening. Postnasal drip deriving from nasal disease is also recognized for promoting vocal cords’ closure [26].

The coexistence of anxiety/depression and DB in individuals is well-documented. Dysfunctional breathing leads to significant alterations in respiratory rate, depth of breathing, and time of breath holding, mainly influenced by existing or past traumatic experiences and psychological conditions such as anxiety [4]. The significance of psychological well-being, particularly in relation to anxiety states, is frequently observed in DB, and this association has been sufficiently strong, leading to the consideration of DB as a somatization illness—a clinical manifestation of mental health issues [1,29,30]. In adults with severe asthma, DB has been associated with combined symptoms of anxiety and depression [31]. In a recent study which was conducted in children and adolescents with functional respiratory disorders, including DB, increased scores for anxious/depressed behavior were reported [32]. 

## 6. Diagnosis of Dysfunctional Breathing

**Figure 2:**
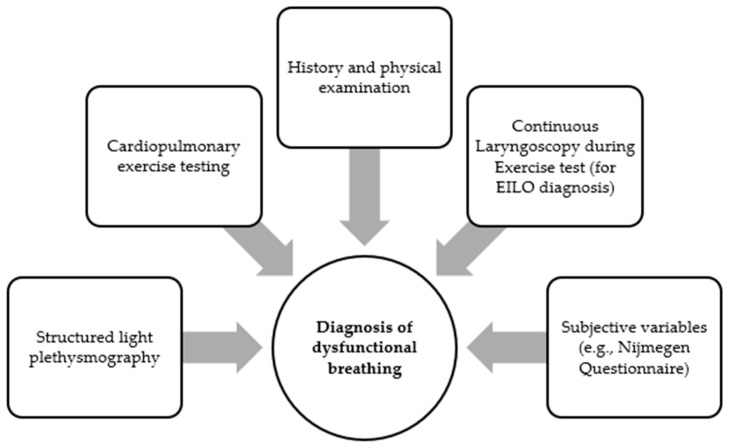
Diagnostic tools for dysfunctional breathing.

Considering the multidimensionality of breathing and the complexity of DB diagnosis, relying on a single criterion is inadequate. On the contrary, a multicomponent assessment is recommended, which includes a detailed history, physical examination, additional diagnostic procedures, and subjective variables [10,33]. Figure 2 epigrammatically illustrates the available tools for the diagnosis of DB. The inconsistent correlation between the range of symptoms experienced and their presumably underlying mechanisms in DB remains poorly comprehended due to the prevailing conceptualization of symptoms. This presents a broader challenge in understanding DB [34]. Differentiating symptoms associated with DB from those arising due to other causes is crucial. This includes unconfirmed respiratory, cardiac, or metabolic conditions leading to breathlessness, poor physical condition, panic disorders with symptoms more clearly connected to direct manifestations of anxiety, and the infrequent incidence of deliberately fabricated or induced illnesses, either by the patient or through proxy during childhood [1]. The most common differential diagnoses of DB include asthma; exercise-induced bronchoconstriction; upper airway obstruction; cardiac diseases; disorders that can cause neuromuscular problems; and reaching physiological limit during exercise [1,6] (see Table 3). The diagnosis of DB should not only rely on the exclusion of organic causes or treating them, but should involve the positive identification of a distinct and clear clinical entity [35]. The detailed information that will derive from a thoroughly gathered history will contribute to reaching DB diagnosis to a significant extent. Children may complain about breathlessness which may manifest at rest, but it mainly occurs during exertion, worsening with rising levels of the duration and intensity of exercise or with anxiety or anticipation [1,6]. Other reported symptoms include noisy breathing, wheeze, and stridor, along with coughing and throat clearing, obstruction, or lump in the throat at episodes, while pain in chest, discomfort, or tightness that are commonly noted may arise from biochemical or biomechanical causes, or be linked with exercise-induced bronchoconstriction or gastro-esophageal reflux [6]. Symptoms of EILO often appear during intense physical activity, unlike exercise-induced bronchoconstriction, where symptoms tend to peak up to 20 min after exercise stops. Dyspnea linked with EILO might be followed by coarse or high-pitched inspiratory breath sounds, sometimes advancing to clear stridor, and this can lead to severe respiratory distress, heightened breathing, panic reactions, and increased ventilatory demands all through the exercise session [36].

The significance of a comprehensive physical examination is frequently undervalued. Nevertheless, the observation of the overall presentation, such as inefficient and excessive upper chest activity, secretions, the use of auxiliary respiratory muscles, an increased breathing rate, signs of anxiety, and lifted shoulders, forms the foundation for making decisions related to the subsequent diagnosis [6,35].

Frequently, history and physical examination alone have been proven insufficient for diagnosing DB. Additional diagnostic procedures should be conducted to confirm the diagnosis. Structured light plethysmography (SLP), which is a light-based non-contact method that captures tidal breathing and analyses of regional differences in chest wall movements, has demonstrated its effectiveness as a useful screening method for cases of DB in children [37]. If DB symptoms are induced by exercise, an exercise challenge test or eucapnic voluntary hyperpnea (EVH) must be performed in order to differentiate exercise–induced bronchoconstriction from EILO [38,39]. Cardiopulmonary exercise testing (CPET) has its own role to the identification and management of DB, and ramp-incremental CPET can be highly beneficial in exploring unexplained dyspnea, enabling the identification of any underlying physiological reasons for exertional breathlessness that may not be evident in tests conducted at rest [40]. CPET offers a non-invasive comprehensive assessment of the cardiovascular, ventilatory, and metabolic responses to exercise and is a powerful diagnostic and prognostic tool [41,42]. The respiratory parameters measured by CPET, such as minute ventilation (V′_E_)/carbon dioxide production (V′_CO2_) slope, ventilatory equivalent for CO_2_ (V_eqCO2_), respiratory exchange ratio, end-tidal carbon dioxide tension, respiratory rate, and tidal volume, are particularly valuable for diagnosing DB [43].

The gold standard method for identifying EILO, although an invasive and distressing experience for children and adolescents, is flexible nasal laryngoscopy. This procedure allows for a clear visualization of the larynx during exercise [1,36]. To enhance the comfort of the frequently young population undergoing examination, it is essential to use a local anesthetic and lubricant. Laryngoscopy at rest can reveal abnormalities in the larynx, glottis, or supraglottic region that may predict laryngeal obstruction during increased inspiratory airflow. However, when pulmonary function tests are normal or treatment for exercise-induced bronchoconstriction is ineffective, a Continuous Laryngoscopy during Exercise test (CLE) may validate the diagnosis of EILO. This test defines the location of obstruction, evaluates its severity, and guides both functional and surgical management [44,45].

It is desirable to use a combination of objective and subjective criteria for identifying DB. Questionnaires are valuable for quantifying and evaluating the normality of subjective sensations [35]. The most frequently used questionnaire for detecting DB is the Nijmegen Questionnaire [1]. The Nijmegen Questionnaire primarily captures the subjective, psychological aspect of breathing and its reactions to stress [35]. It was implemented nearly 40 years ago to identify patients with hyperventilation complaints, and although it lacks validation for use in children and adolescents, it has been employed in several pediatric studies and can be beneficial for clinical decisions and appraising treatment as part of a comprehensive assessment [8,11,33,46]. This questionnaire consists of 16 questions, with the frequency of occurrence to be expressed on a five-point ordinal scale (1 = never, 5 = very frequently), while these complaints cover various systems such as (a) cardiovascular; (b) neurological; (c) respiratory; (d) gastrointestinal; and (e) psychological. The Nijmegen Questionnaire demonstrated a 91% sensitivity and 95% specificity in association with clinical diagnosis [46]. Scoring above 23 is linked with HVS, although this calculation is based on a positive hyperventilation provocation test as the most widely accepted method, which is no longer considered as credible for HVS diagnosis [5]. Another questionnaire that developed with the aim to diagnose HVS in children is the Hyperventilation Syndrome Ambroise-Pare’Enfant (SHAPE), which involves a simplified 17-item questionnaire, although the lack of validation in children constitutes its main limitation [47].

## 7. Therapeutic Approach to Dysfunctional Breathing

**Figure 3:**
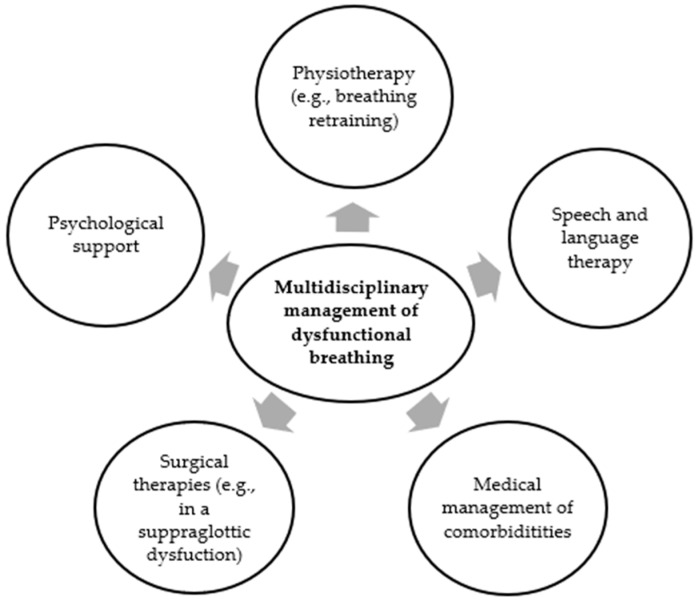
The multidisciplinary management of dysfunctional breathing.

A comprehensive evaluation of DB and its care approaches necessitates a multidisciplinary assessment and treatment [18]. Most patients with DB can be effectively handled through a positive diagnosis, reassurance, disease-specific advice, and breathing retraining [48]. However, there is limited information in the existing literature concerning the management of pediatric patients with DB, and this can be attributed to variations among studies in terms of the recruitment methods used, as there are currently a lack of standardized diagnostic tools for DB [4]. Furthermore, the coexistence of DB with other associated conditions, such as asthma, GERD, nasal disease, or anxiety, complicates matters, as these conditions should also be diagnosed and treated [1]. The involvement of multidisciplinary team members, including a respiratory physician; physiotherapist; clinical nurse; specialist speech and language therapist; and psychologist, is essential for managing diverse symptoms and entities that may demand different therapeutic approaches [18,48]. In some cases, patients may benefit from the input from more than one of these disciplines. Therapy should be in accordance with an individualized assessment, goal setting, and the development of a care plan in collaboration with the patients and their families [1]. Figure 3 depicts the multidisciplinary management of DB.

Non-pharmacological interventions have become prominent in the treatment of DB. The most frequently used intervention for DB is breathing retraining exercises. Physiotherapy strives to diminish and prevent symptoms of DB by guiding patients to identify their own pathological breathing patterns, retrain their physiological breathing, and use well-directed self-help techniques. Providing education and information about these measures is of considerable significance for therapy [48]. Clinical experience, along with feedback from patients and their families, suggest that young individuals with DB witness substantial improvements in both physical health and quality of life after undergoing physiotherapy, where breathing retraining is the essential element of this treatment [49]. The main therapeutic goal is to retrain physiological breathing, with a particular emphasis on diaphragmatic breathing that is taught while the patient is positioned horizontally, vertically, and finally during physical activity, along with the inclusion of interventions for addressing tension in a strained abdominal wall. Longer-term teaching of relaxation techniques can contribute to the therapeutic effect [48]. Nevertheless, a previous systematic review that aimed to determine whether breathing retraining has beneficial effects on children and adolescents with DB reported that it is unclear whether these therapies considered beneficial for this patient group or if specific types of breathing exercises were superior over others [50]. However, the findings of a recent study indicated that a multifaceted intervention comprising physiotherapy which included diaphragmatic breathing, elements of cognitive behavioral therapy, and a tailored rehabilitation plan might eliminate symptoms of inspiratory distress in adolescent athletes with EILO [51].

Various other interventions include glottic retraining, botulinum toxin injections, low-dose amitriptyline, and inspiratory muscle strength training devices that have been associated with symptom reductions in adults and adolescents with VDC [52]. The use of inhaled anticholinergic agents may be a part of EILO therapy, as these agents seem to reduce the muscarinic stimulus of laryngeal adductors and may positively alter upper airway secretions [53]. Nonetheless, a recent randomized crossover trial that was conducted on adolescents and young adults aged 12–25 years, aiming to examine the effects of inhaled ipratropium bromide in EILO diagnosed by a CLE test and classified by CLE scores, supported no improvement in CLE score, dyspnea, or exercise capacity in subjects with EILO and did not recommend ipratropium bromide for the treatment of EILO [53].

Alternatively, surgical therapies could be considered in cases where a supraglottic component to the EILO exists, and supraglottoplasty may find a role. Additionally, adenoidectomy and turbinate reduction could be beneficial in cases such as adenoidal hypertrophy and rhinitis, although they are typically initially addressed with intranasal steroid therapy [1,54].

Speech and language therapy is essential for children and adolescents with upper airway dysfunction. A speech and language therapist can provide a specialized evaluation for upper airway dysfunction, contributing to a thorough differential diagnosis and non-pharmacological management. They can offer laryngoscopy services for provocation testing and implement personalized therapy programs with the goal of resolving upper airway symptoms to a premorbid state or maximal potential [18]. Newson et al., who aimed to audit a care pathway for children and adolescents with DB, particularly focusing on ILO and EILO, referred the care pathway to specialist voice speech and language therapy (SALT). They showed that most participants had good control of their DB, specifically in terms of school attendance and ability to participate in activities, with confidence in their ability to control their DB symptoms. This demonstrated an improved morbidity after SALT assessment and the implementation of a treatment protocol [13].

In the case of DB, psychological assessment and support for the patient are equally significant, especially in the presence of a psychiatric condition (e.g., anxiety/depression) or if the symptoms of DB cause stress-inducing symptoms. Examining common psychological stressors can be beneficial. For some individuals, depending on the severity of pre-existing anxieties or those caused by this condition, formal psychological support may be required. If a more significant psychosocial disturbance is identified, it should be addressed in the appropriate way [55]. A clinical psychologist may assess and formulate an illustration of the psychological effects of respiratory disease. They can facilitate patients and their families to understand the nature and implications of their respiratory condition. Additionally, they enable patients to set therapy goals while addressing unrealistic expectations. They empower patients to adapt to their respiratory condition by exploring ways to decrease its effect and foster self-efficacy and coping abilities. Moreover, they manage any fears, concerns, or misunderstandings about the respiratory diagnosis and assist patients and their families in coping with the emotional triggers of breathing difficulties, such as anxiety or depression [18].

## 8. Conclusions

This review enhances the comprehension of DB in children through the existing literature, highlighting its classifications, comorbidities, available diagnostic methods, and therapeutic options. A detailed patient’s assessment based on a combination of history taking, physical examination, diagnostic procedures, and comorbidity detection may lead to a precise and prompt DB diagnoses that will determine the appropriate therapy, preventing the use of unnecessary treatments. However, the complex nature of DB, requiring a multidimensional approach, combined with the lack of studies on children, further complicates the understanding and management of this specific condition. Therefore, further research conducted through high-quality studies is necessary to substantiate evidence in this field. This includes standardizing diagnostic tools for DB in the pediatric population, establishing therapeutic approaches, and optimizing clinical outcomes via individualized treatment plans in this age group.

## Figures and Tables

**Table 1:** Proposed classifications of dysfunctional breathing.

Author	Classification of DB
Barker and Everald [3]	Thoracic DB (T-DB) Functional T-DB (e.g., pattern disordered breathing) Structural T-DB (e.g., phrenic nerve palsy)
	Extrathoracic DB (ET-DB) Functional ET-DB (e.g., vocal cord dysfunction) Structural ET-DB (e.g., subglottic stenosis)
Boulding et al. [5]	Hyperventilation syndrome
	Periodic deep sighing
	Thoracic dominant breathing
	Forced abdominal expiration
	Thoraco-abdominal asynchrony
Barker et al. [1]	Breathing Pattern Disorders (BPD) Hyperventilation Syndrome Subcategories of BPD
	Inducible laryngeal obstruction Vocal cord dysfunction Supraglottic collapse

DB: Dysfunctional breathing.

**Table 3:** Main differential diagnoses of dysfunctional breathing.

Differential Diagnoses
Asthma
Upper airway obstruction (e.g., laryngomalacia)
Exercise-induced bronchoconstriction
Cardiac diseases (e.g., exercise-induced tachyarrhythmia)
Disorders that can cause neuromuscular problems (e.g., pectus excavatum or carinatum, scoliosis)
Reaching physiological limit during exercise

## Data Availability

Not applicable.
